# Integrated Multi-Criteria Decision-Making for the Selection of Natural and Synthetic Fiber-Reinforced Composites for Unmanned Aerial Vehicle Micro-Turbojet Engine Inlets

**DOI:** 10.3390/polym18162027

**Published:** 2026-08-21

**Authors:** Abderraouf Gherissi

**Affiliations:** Mechanical Engineering Department, Faculty of Engineering, University of Tabuk, P.O. Box 741, Tabuk 71491, Saudi Arabia; a.gresi@ut.edu.sa

**Keywords:** natural fiber-reinforced polymer composites (NFRPCs), synthetic fiber-reinforced polymer composites (SFRPCs), micro-turbojet engine inlet, unmanned aerial vehicle (UAV), multi-criteria decision-making (MCDM), AHP-TOPSIS, sustainable aerospace materials

## Abstract

This study develops an integrated Analytic Hierarchy Process (AHP) and Technique for Order Preference by Similarity to Ideal Solution (TOPSIS) multi-criteria decision-making (MCDM) framework to systematically evaluate and rank composite material combinations based on 24 fibers (16 natural and 8 synthetic), 15 matrices (thermosets, thermoplastics, and biopolymers), and 9 fiber volume fractions (30–70%) for UAV inlet applications. Ten evaluation criteria covering technical performance, environmental sustainability, and economic viability were weighted using AHP pairwise comparisons based on Saaty’s 1–9 scale, yielding a consistency ratio of CR = 0.009, which confirms the reliability of the judgments. The TOPSIS analysis identified Carbon (PAN-HM)/Epoxy as the optimal composite material, achieving the highest TOPSIS score of 0.8893. In contrast, Flax/Epoxy emerged as the best natural fiber composite, with a TOPSIS score of 0.2686, indicating a performance gap of approximately 231% in favor of the synthetic composite. Comprehensive sensitivity analysis across four weighting scenarios (Equal, Technical, Environmental, and Economic) confirmed the stability of the reinforcement rankings, with Carbon (PAN-HM) remaining the top synthetic fiber and flax the top natural fiber across all scenarios. The findings contribute to the growing body of knowledge on sustainable aerospace materials and provide practical guidance for UAV designers seeking to optimize material selection for micro-turbojet engine inlet components, supporting the development of more environmentally responsible UAV designs while maintaining the performance requirements for safe and reliable operation.

## 1. Introduction

Unmanned aerial vehicles (UAVs) have experienced rapid growth in civilian, industrial, and military applications, including surveillance, inspection, logistics, precision agriculture, and emergency response. This expansion has increased the demand for compact propulsion systems capable of delivering high thrust, fuel efficiency, and reliable operation under varying flight conditions. Micro turbojet engines (MTJEs) have emerged as attractive propulsion systems because of their high thrust-to-weight ratio, compact configuration, and ability to operate over a wide range of operating conditions. Recent thermodynamic and performance analyses have demonstrated that MTJE efficiency is strongly influenced by engine operating conditions and component design, particularly the inlet section, which governs airflow quality entering the compressor and directly affects engine efficiency and stability [1,2,3,4,5].

The inlet is one of the most critical structural components of a micro turbojet engine because it is continuously subjected to aerodynamic loading, vibration, thermal gradients, and cyclic mechanical stresses during operation. Consequently, material selection for the inlet requires an appropriate balance between mechanical strength, fatigue resistance, thermal stability, low density, manufacturability, and environmental performance. Classical composite laminate theory has become an effective tool for predicting the structural response of laminated composite materials used in aerospace structures, while thermal analysis remains essential for evaluating heat transfer and temperature distribution within engine components [6,7].

Fiber-reinforced polymer composites have become increasingly attractive for aerospace applications because of their high specific strength, corrosion resistance, and excellent fatigue performance. Synthetic fiber composites such as carbon, glass, and aramid fiber-reinforced polymers have been widely adopted owing to their superior structural performance under demanding service conditions [8,9,10,11]. However, the production of synthetic fibers is energy intensive and contributes significantly to greenhouse gas emissions and end-of-life waste, motivating the search for more sustainable composite alternatives [11,12,13].

Natural fiber-reinforced polymer composites (NFRPCs) have emerged as promising lightweight materials that combine acceptable mechanical properties with reduced environmental impact. Comprehensive reviews have shown that flax, kenaf, abaca, banana, and other lignocellulosic fibers provide low density, competitive specific mechanical properties, lower embodied energy, and reduced carbon emissions compared with conventional synthetic composites [10,11,12].

Recent research has demonstrated the potential of natural fiber composites for aerospace structures. Vinayagam et al. [2] evaluated the thermal and structural behavior of natural fiber composites for UAV microjet engine nozzles and reported that several natural fiber systems could withstand representative thermal and structural loading conditions while offering considerable environmental advantages. Likewise, recent studies have highlighted the growing use of natural fiber composites in aircraft structures and cabin components, emphasizing their capability to reduce structural weight and carbon emissions without significantly compromising mechanical performance [13,14,15,16].

Nevertheless, the application of natural fiber composites to MTJE inlet structures remains largely unexplored, particularly when considering simultaneous structural, thermal, economic, and environmental requirements.

Selecting the most suitable composite material for aerospace applications is inherently a multi-criteria decision problem because numerous conflicting factors must be considered simultaneously. Mechanical properties alone are insufficient for material selection, as thermal conductivity, thermal stability, fatigue life, density, cost, carbon footprint, and biodegradability also influence overall performance [14].

Multi-criteria decision-making (MCDM) techniques can be applied to material selection problems. And any systematic material selection problem using MCDM methods must be structured around three fundamental pillars: Alternatives, Criteria, and Weightage [17,18,19,20,21].

The alternatives represent the candidate materials or material configurations, while the criteria describe the technical, economic, environmental, and operational requirements used to evaluate their suitability [17]. Since these criteria do not necessarily have equal importance, an appropriate weighting method is required before ranking the alternatives [18,19]. Several MCDM approaches can be employed for this purpose, including AHP, graph-theory-based methods, TOPSIS, and fuzzy MCDM techniques [19,21].

AHP provides a systematic procedure for determining criteria weights through pairwise comparisons and has been further developed to improve the consistency of expert judgments [17]. TOPSIS ranks alternatives according to their relative proximity to the ideal and negative-ideal solutions, making it suitable for problems involving multiple conflicting criteria [18,21]. Fuzzy MCDM approaches can incorporate uncertainty and imprecision in expert judgments and have been applied to complex engineering selection problems [19], while the choice of normalization and weighting procedures can significantly influence the resulting rankings [20].

The Analytic Hierarchy Process (AHP) and the Technique for Order Preference by Similarity to the Ideal Solution (TOPSIS), have become powerful tools for engineering material selection because they enable systematic evaluation of multiple competing criteria. Recent developments have further improved AHP consistency and TOPSIS robustness, making these methods increasingly suitable for sustainable engineering applications [17,18,21]. In the present study, the alternatives are the investigated natural and synthetic fiber-reinforced polymer composites, while the criteria represent their mechanical, thermal, economic, and environmental performance. AHP-TOPSIS was selected because it provides a clear separation between criteria weighting and alternative ranking.

Despite the growing interest in sustainable aerospace materials, few studies have simultaneously compared synthetic and natural fiber-reinforced polymer composites for MTJE inlet applications using a comprehensive decision-making framework that integrates mechanical, thermal, environmental, and economic criteria. Furthermore, the robustness of material rankings under varying design priorities has received limited attention.

Therefore, this study develops an integrated AHP–TOPSIS framework to evaluate natural and synthetic fiber-reinforced polymer composites for MTJE inlet applications. Ten evaluation criteria, including tensile strength, thermal stability, thermal conductivity, material cost, carbon footprint, and biodegradability, are considered. The robustness of the material rankings is further examined through four sensitivity scenarios representing technical, environmental, economic, equal-weight, and balanced priorities. The proposed framework provides a systematic methodology for identifying lightweight and sustainable composite materials capable of satisfying the demanding structural and thermal requirements of micro turbojet engine inlet systems while supporting environmentally responsible aerospace design.

## 2. Materials and Methods

**Figure 1:**
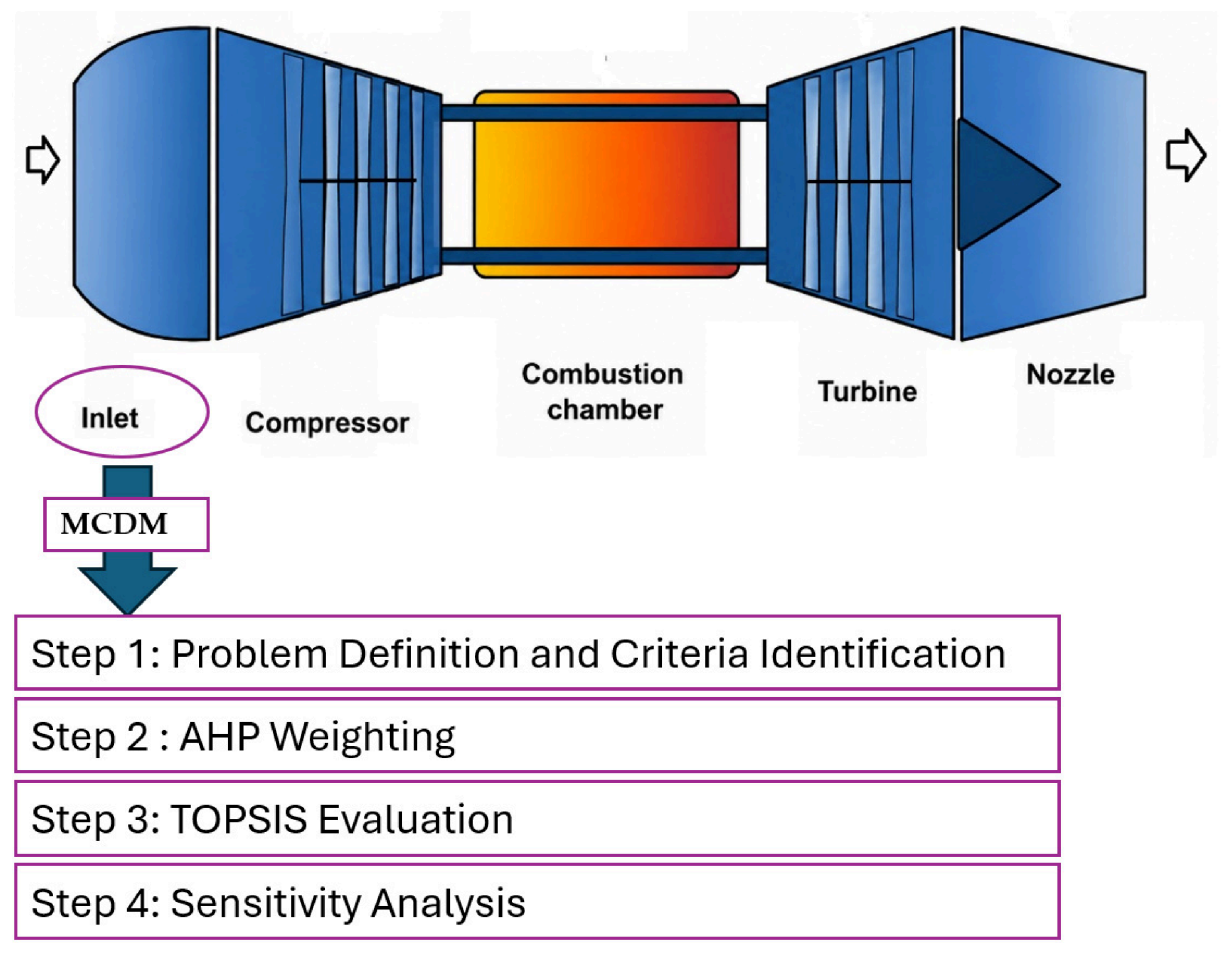
Schematic of the MTJE Components and Material Selection Procedure for the Inlet Using MCDM.

The air intake system of a micro-turbojet engine constitutes the initial interface between the freestream airflow and the engine core, serving the critical function of capturing and delivering air to the compressor with minimal losses [1]. The intake, positioned upstream of the compressor, decelerates the incoming airflow, thereby increasing its static pressure through a ram compression effect [2]. Figure 1 presents a schematic of the micro-turbojet engine components and the material-selection procedure for the inlet using MCDM.

This study is based on the study of, Balli Ö [3], a MTJE adopted in this study is a commercially available small-scale gas turbine designed for UAV and drone applications, producing a maximum thrust of 125 N at sea-level static conditions [3]. The engine operates on JP-8 jet fuel with a lower heating value of 42,800 kJ/kg. The engine was tested at four distinct operation modes corresponding to rotational speeds as shown in Table 1 [3].

Table 2 summarizes the inlet flow conditions and associated performance parameters at Inlet Station [3] across four operational modes (Mode-1 through Mode-4). The inlet temperature (T_1_) remains constant at 288.15 K for all modes, while the inlet pressure (P_1_) is likewise fixed at 101.325 kPa, indicating that the engine operates under steady, near-standard ambient conditions. The air mass flow rate (ṁ_1_) increases progressively from 0.1324 kg/s in Mode-1 to 0.2412 kg/s in Mode-4, reflecting a monotonic rise in engine throughput. The specific heat capacity of air (cP,air) and the specific gas constant (R_air) are held constant at 1.005 kJ/kg·K and 0.287 kJ/kg·K, respectively, across all modes, consistent with the assumption of calorically perfect gas behavior. The reference temperature (T_0_) is also maintained at 288.15 K throughout. These data collectively illustrate that the inlet section operates under thermally and pressure-wise uniform conditions, with variations in performance driven primarily by changes in mass flow rate, rather than by fluctuations in inlet thermodynamic state.

### 2.1. Inlet Equations for Micro Turbojet Engine Operating Conditions

Constitutive relationships and material response models govern the inlet structure under the engine’s operational envelope. Inlet Aerodynamic and Thermodynamic Equations at constant pressure, the specific heat capacity of air is temperature-dependent [4], as expressed in Equation (1):

(1) $$c_{P,air}(T)=1.04841-3.83719\times 10^{-4}T+9.45378\times 10^{-7}T^{2}-5.49031\times 10^{-10}T^{3}+7.92981\times 10^{-14}T^{4}$$

where the temperature is evaluated in K.

Specific Heat Ratio for Air expressed in Equation (2):

(2) $$k_{air}=\frac{1}{1-\frac{R_{air}}{c_{P,air}}}=\frac{c_{P,air}}{c_{P,air}-R_{air}}$$

For air at inlet T = 288.15 K, $k_{air}=1.4$ [3].

The energy rate at the inlet is calculated [3], as expressed in Equation (3):

(3) $${\dot{E}}_{1}={\dot{m}}_{1}\left(c_{P,1}T_{1}-c_{P,0}T_{0}\right)$$

The exergy rate of air at the inlet is estimated by [3], as expressed in Equation (4):

(4) $${\dot{E}}_{X1}={\dot{m}}_{1}\left[(c_{P,1}T_{1}-c_{P,0}T_{0})-c_{P,0}T_{0}ln\left(\frac{T_{1}}{T_{0}}\right)+R_{air}T_{0}ln\left(\frac{P_{1}}{P_{0}}\right)\right]$$

The air mass flow rate through the inlet can be related to the engine operating conditions using Equation (5):

(5) $${\dot{m}}_{1}={\dot{m}}_{1,ref}\left(\frac{P_{1}}{P_{0}}\right)\sqrt{\frac{T_{0}}{T_{1}}}$$

The momentum equation at the inlet can be expressed as:

(6) $$F={\dot{m}}_{1}V_{1}+(P_{1}-P_{0})A_{1}$$

For subsonic inlet conditions, the velocity at the inlet face is:

(7) $$V_{1}=M_{1}\sqrt{k_{air}R_{air}T_{1}}$$

where $M_{1}$ is the Mach number at the inlet face.

The heat flux through the inlet wall is determined by Fourier’s law, as expressed in Equation (8):

(8) $$q_{w}=-k_{m}\frac{\partial T}{\partial x}{|}_{wall}$$

where

$q_{w}$ = heat flux at the wall (W/m^2^)

$k_{m}$ = thermal conductivity of the material (W/m·K)

∂T/∂x = temperature gradient at the wall (K/m)

The convective heat transfer coefficient can be determined from Nusselt number correlations [6]:

(9) $$Nu=\frac{hL}{k_{f}}$$

For turbulent flow in a pipe, the Dittus-Boelter correlation [7] applies Equation (10):

(10) $$Nu=0.023Re^{0.8}Pr^{0.4}$$

where:

$Re=\frac{\rho VD}{\mu }$ = Reynolds number

$Pr=\frac{\mu c_{p}}{k_{f}}$ = Prandtl number

h = convective heat transfer coefficient (W/m^2^·K)

L = characteristic length (m)

$k_{f}$ = thermal conductivity of fluid (W/m·K)

The thermal stress induced in the inlet wall material is governed by Equation (11) [8]:

(11) $$\sigma_{th}=\frac{E_{m}\alpha_{m}(T_{w}-T_{\infty })}{1-\nu_{m}}$$

where:

$\sigma_{th}$ = thermal stress (MPa)

$E_{m}$ = elastic modulus of material (MPa)

$\alpha_{m}$ = coefficient of thermal expansion (1/K)

$T_{w}$ = wall temperature (K)

$T_{\infty }$ = freestream temperature (K)

$\nu_{m}$ = Poisson’s ratio

The total stress in the inlet wall under combined loading is as follow:

(12) $$\sigma_{total}=\sigma_{th}+\sigma_{mech}$$

where $\sigma_{mech}$ is the mechanical stress due to pressure loading.

**Figure 2:**
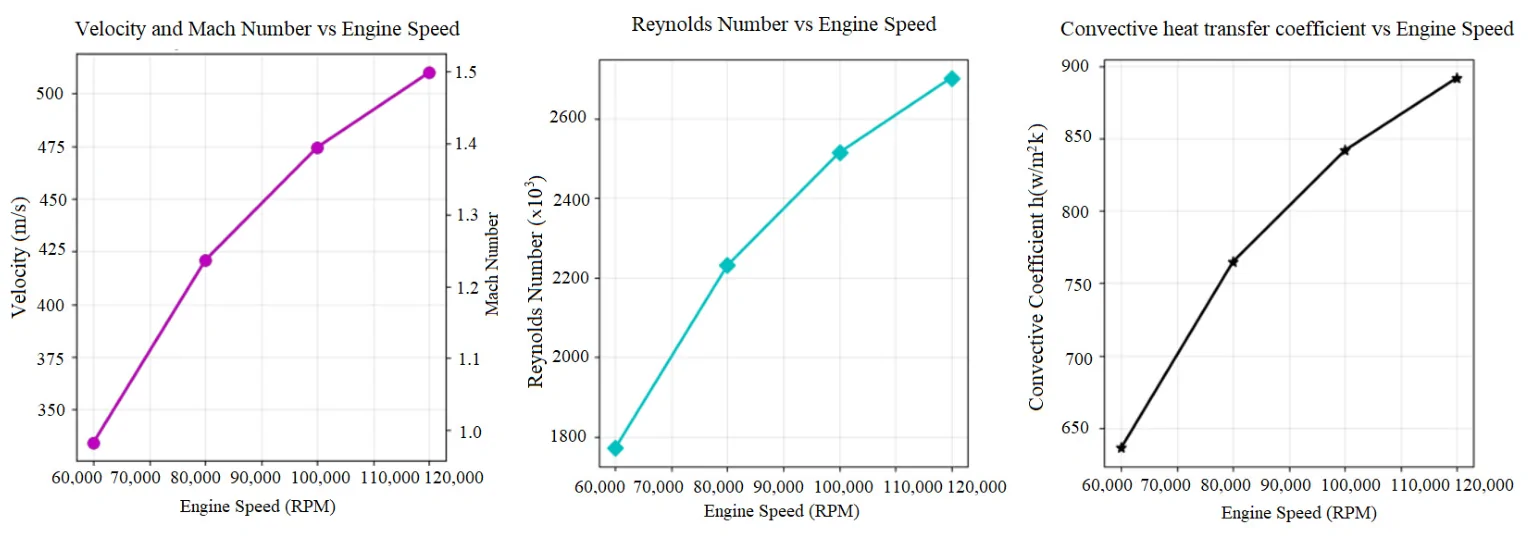
Simulated aerodynamic and heat transfer characteristics of the micro-turbojet engine (MTJE) inlet at different operating speeds.

Based on the simulation presented in Figure 2 for the MTJE inlet, the increasing thermal and aerodynamic loads present both opportunities and challenges for the application of NFRPC/SFRPC as inlet materials. The main opportunity lies in their low density and favorable specific stiffness, which could reduce the overall engine weight, a key advantage for UAV applications. Their damping properties may also help reduce vibration-induced stresses.

However, several challenges must be considered. High-velocity airflow generates significant convective heat transfer, which may approach the thermal degradation limits of the fibers and matrix, leading to matrix softening, fiber degradation, and loss of mechanical integrity over time. In addition, mechanical stresses caused by pressure loading, combined with thermal stresses resulting from temperature gradients across the inlet wall, may exceed the strength and creep resistance of these materials, particularly at higher engine speeds where thermal loads become more severe. Furthermore, moisture absorption, anisotropic behavior, and manufacturing variability may affect the reliability and long-term performance of NFRPC/SFRPC materials under the harsh and cyclic operating conditions of a turbojet engine.

### 2.2. Inlet Material Performance Equations for NFRPC/SFRPC

The thermal conductivity of NFRPC/SFRPC materials at inlet conditions can be predicted using the rule of mixtures, Equation (13):

(13) $$k_{c}=k_{f}V_{f}+k_{m}V_{m}$$

where:

$k_{c}$ = composite thermal conductivity (W/m·K)

$k_{f}$ = fiber thermal conductivity (W/m·K)

$k_{m}$ = matrix thermal conductivity (W/m·K)

$V_{f}$ = fiber volume fraction

$V_{m}$ = matrix volume fraction

The heat transfer rate through the inlet wall is calculated using Equation (14).

(14) $$\dot{Q}=\frac{k_{c}A}{t}(T_{w}-T_{\infty })$$

where:

$\dot{Q}$ = heat transfer rate (W)

$k_{c}$ = composite thermal conductivity (W/m·K)

A = wall surface area (m^2^)

t = wall thickness (m)

$T_{w}$ = wall temperature (K)

$T_{\infty }$ = freestream temperature (K)

The thermal resistance of the inlet wall is expressed by Equation (15).

(15) $$R_{th}=\frac{t}{k_{c}A}$$

The steady-state temperature distribution through the inlet wall expressed by Equation (16).

(16) $$T(x)=T_{w}-\frac{\dot{Q}}{k_{c}A}x$$

where x is the distance from the inner wall.

The Thermal Diffusivity of NFRPC expressed by Equation (17) [9].

(17) $$\alpha_{th}=\frac{k_{c}}{\rho_{c}c_{p,c}}$$

where:

$\alpha_{th}$ = thermal diffusivity (m^2^/s)

$\rho_{c}$ = composite density (kg/m^3^)

$c_{p,c}$ = specific heat of composite (J/kg·K)

The pressure force on the inlet wall is calculated using Equation (18):

(18) $$F_{p}=(P_{2}-P_{1})A$$

where $P_{2}$ is the compressor inlet pressure.

The wall stresses due to pressure loading in the cylindrical inlet section are calculated using Equations (19) and (20) [8].

(19) $$\sigma_{hoop}=\frac{(P_{2}-P_{1})D}{2t}$$

(20) $$\sigma_{axial}=\frac{(P_{2}-P_{1})D}{4t}$$

where:

$\sigma_{hoop}$ = hoop stress (MPa)

$\sigma_{axial}$ = axial stress (MPa)

D = inlet diameter (m)

t = wall thickness (m)

The Von Mises Equivalent Stress is calculated using Equation (21):

(21) $$\sigma_{vonMises}=\sqrt{\sigma_{hoop}^{2}+\sigma_{axial}^{2}-\sigma_{hoop}\sigma_{axial}}$$

Safety Factor is calculated using Equation (22):

(22) $$SF=\frac{\sigma_{ultimate}}{\sigma_{vonMises}}$$

where $\sigma_{ultimate}$ is the ultimate tensile strength of the material.

### 2.3. Mechanical Properties of NFRPC/SFRPC

The mechanical properties of NFRPC/SFRPC are determined by the fiber characteristics, matrix properties, fiber-matrix interfacial adhesion, and processing parameters [10,11,12,13,14,15].

Table 3 presents the mechanical properties of candidates’ natural fibers and synthetic fibers.

The material-property data presented in Table 3 and Table 4 were compiled from multiple published literature sources to provide a representative range of the reported properties for each composite material. The use of ranges rather than single values accounts for the variability associated with differences in material grades and formulations, fiber source and quality, fiber volume fraction, manufacturing and processing conditions, testing standards, specimen preparation, measurement techniques, and experimental uncertainty. This approach provides a broader representation of the material behavior reported in the literature and avoids assigning a single value that may not represent the variability of a given composite system. The reported ranges should therefore be interpreted as literature-based property intervals used for comparative MCDM analysis rather than as fixed or universal material properties.

### 2.4. Multi-Criteria Decision-Making (MCDM) Using the Integrated AHP-TOPSIS Framework

The selection of optimal NFRPC/SFRPC reinforced polymer composites for UAV micro turbojet engine inlet applications requires systematic evaluation of multiple, often conflicting, performance criteria [14,16]. Multi-Criteria Decision-Making (MCDM) methodologies, particularly the Analytic Hierarchy Process (AHP) [14,17] and the technique for order Preference by Similarity to Ideal Solution (TOPSIS), provide robust frameworks for integrating environmental, economic, technical, and social dimensions into material selection decisions. The integrated AHP-TOPSIS approach enables quantitative ranking of alternatives while accounting for performance trade-offs [17,21].

The AHP methodology [16], structures complex decision problems into hierarchical levels comprising the overall goal, evaluation criteria, and alternative materials. The hierarchical structure enables systematic pairwise comparisons to determine criteria weights and alternative priorities [16,17].

For this study, the hierarchy comprises three levels:

Level 1: defines the overall objective, selecting the optimal NFRPC/SFRPC for the inlet zone of a UAV micro-turbojet engine.

Level 2: establishes the evaluation criteria, which encompasses mechanical, thermal, physical, economic, and environmental performance. Finally,

Level 3: lists the candidate materials, including both natural fiber composites and conventional alternatives [16]

The relative importance of criteria is established through pairwise comparisons using Saaty’s fundamental scale [16]:

$$s_{ij}=\left\{\begin{array}{cc} 1 & \mathrm{if}\;i\;\mathrm{and}\;j\;\mathrm{are}\;\mathrm{equally}\;\mathrm{important}\\ 3 & \mathrm{if}\;i\;\mathrm{is}\;\mathrm{moderately}\;\mathrm{preferred}\;\mathrm{to}\;j\\ 5 & \mathrm{if}\;i\;\mathrm{is}\;\mathrm{strongly}\;\mathrm{preferred}\;\mathrm{to}\;j\\ 7 & \mathrm{if}\;i\;\mathrm{is}\;\mathrm{very}\;\mathrm{strongly}\;\mathrm{preferred}\;\mathrm{to}\;j\\ 9 & \mathrm{if}\;i\;\mathrm{is}\;\mathrm{absolutely}\;\mathrm{preferred}\;\mathrm{to}\;j \end{array}\right.$$

Intermediate values (2, 4, 6, 8) are used for compromise judgments. The pairwise comparison matrix is constructed as [16]:

(23) $$S=\left[\begin{array}{cccc} 1 & s_{12} & \ldots & s_{1k}\\ 1/s_{12} & 1 & \ldots & s_{2k}\\ \vdots & \vdots & \ddots & \vdots \\ 1/s_{1k} & 1/s_{2k} & \ldots & 1 \end{array}\right]$$

where k is the number of criteria and $s_{ji}=1/s_{ij}$.

The geometric mean method is employed to derive criteria weights [16]:

As

(24) $$R_{i}={\left(\prod_{j=1}^{k}s_{ij}\right)}^{1/k}$$

(25) $$w_{i}=\frac{R_{i}}{\sum_{i=1}^{k}R_{i}}$$

where $g_{i}$ is the product of pairwise comparisons for criterion i, $R_{i}$ is the geometric mean, and $w_{i}$ is the normalized weight [16]. The weight vector is expressed as: $W=[w_{1},w_{2},\ldots ,w_{k}{]}^{T}$, with the constraint $\sum_{i=1}^{k}w_{i}=1$.

The consistency ratio (CR) is calculated to validate the pairwise comparison judgments [17]:

(26) $$CR=\frac{CI}{RI}$$

where CI is the consistency index and RI is the random consistency index [17]. The consistency index is determined as:

(27) $$CI=\frac{\lambda_{max}-k}{k-1}$$

where $\lambda_{max}$ is the maximum eigenvalue of the pairwise comparison matrix.

k: the size of the pairwise comparison matrix

The Technique for Order Preference by Similarity to the Ideal Solution (TOPSIS) ranks alternatives according to their geometric distances from the Positive Ideal Solution (PIS) and Negative Ideal Solution (NIS). The optimal alternative is closest to the PIS and farthest from the NIS [18,19].

The decision matrix $X=[x_{ij}]$ with m alternatives and n criteria is normalized using the vector normalization method [20]:

(28) $$r_{ij}=\frac{x_{ij}}{\sqrt{\sum_{i=1}^{m}x_{ij}^{2}}}$$

where $r_{ij}$ is the normalized performance value of alternative i with respect to criterion j.

The normalized values are multiplied by the AHP-derived criterion weights [20]:

(29) $$v_{ij}=w_{j}\times r_{ij}$$

where $v_{ij}$ is the weighted normalized value and $w_{j}$ is the weight of criterion j.

The positive ideal solution (PIS) and negative ideal solution (NIS) are defined as [21]:

(30) $$A^{+}=\{v_{1}^{+},v_{2}^{+},\ldots ,v_{n}^{+}\}=\{(\underset{i}{max}\;v_{ij}|j\in J),(\underset{i}{min}\;v_{ij}|j\in J^{\prime })\}\;A^{-}=\{v_{1}^{-},v_{2}^{-},\ldots ,v_{n}^{-}\}=\{(\underset{i}{min}\;v_{ij}|j\in J),(\underset{i}{max}\;v_{ij}|j\in J^{\prime })\}$$

where J represents benefit criteria (higher values preferred) and $J^{\prime }$ represents cost criteria (lower values preferred).

The separation of each alternative from the PIS and NIS is calculated using Euclidean distance [21]:

(31) $$D_{i}^{+}=\sqrt{\sum_{j=1}^{n}(v_{ij}-v_{j}^{+}{)}^{2}}$$

(32) $$D_{i}^{-}=\sqrt{\sum_{j=1}^{n}(v_{ij}-v_{j}^{-}{)}^{2}}$$

where $D_{i}^{+}$ is the distance from the PIS and $D_{i}^{-}$ is the distance from the NIS [21].

The relative closeness coefficient ($C_{i}^{*}$) is calculated as [21]:

(33) $$C_{i}^{*}=\frac{D_{i}^{-}}{D_{i}^{+}+D_{i}^{-}}$$

where $0\leq C_{i}^{*}\leq 1$. Higher values indicate better performance [21]. Alternatives are ranked in descending order of $C_{i}^{*}$, with the highest value representing the optimal choice [21].

### 2.5. Technique for Order Preference by Similarity to the Ideal Solution (TOPSIS)

The integrated AHP-TOPSIS framework for selecting optimal NFRPC/SFRPC for UAV inlet applications follows a systematic workflow [16]:

Phase 1: Problem Definition and Criteria Identification

Define decision context: Selection of NFRPC/SFRPC materials for micro turbojet engine inlet components. Identify relevant criteria across three sustainability dimensions [16]. Table 5 shows the dimensions and the criteria for the present study.

**Phase 2: AHP Weighting**

Conduct expert pairwise comparisons for criteria

Calculate normalized weights with consistency verification

Generate stakeholder-specific weight profiles

**Phase 3: TOPSIS Evaluation**

Compile performance data for alternative materials

Normalize decision matrix

Apply AHP weights to create weighted normalized matrix

Identify PIS and NIS for each criterion

Calculate separation measures and closeness coefficients

Rank alternatives

**Phase 4: Sensitivity Analysis**

Conduct sensitivity analysis to assess ranking stability

Examine the influence of weight variations on final rankings

Identify robust alternatives

### 2.6. Application to UAV Inlet NFRPC/SFRPC Selection

For the specific case of UAV micro turbojet engine inlet zone, the evaluation criteria derived from the AHP reflect the operational requirements and performance priorities [16]. The typical weight distribution for inlet applications is presented in Table 6.

Expert judgment-based AHP is one of the widely used MCDM methods for material selection and decision-making problems. Its main advantage is its ability to decompose a complex decision problem into a hierarchical structure of criteria and alternatives, thereby facilitating a systematic evaluation of competing options [31]. The Analytic Hierarchy Process (AHP) provides a general framework for measurement by establishing priorities through pairwise comparisons. These comparisons are performed using numerical judgments based on Saaty’s fundamental absolute scale [32].

AHP has been applied in combination with other MCDM techniques, including Complex Proportional Assessment and the Technique for Order Preference by TOPSIS, for selecting suitable materials for aircraft components such as wings and nose structures. In these studies, AHP was used to determine the relative importance and weights of the evaluation criteria before applying the subsequent material-ranking methods [33].

The reliability of AHP pairwise comparisons depend on the selection of appropriate experts whose knowledge and experience correspond to the scope and objectives of the study. Previous research indicates that the number of experts participating in AHP-based evaluations can vary from 3 to 25, depending on the complexity and requirements of the decision problem [34]. In the present study, the pairwise comparisons were conducted by a panel of 11 experts with combined expertise in composite materials and manufacturing, UAV propulsion systems and aerodynamics, sustainable materials, and life-cycle assessment. The composition of the expert panel was selected to provide multidisciplinary judgment covering the thermal, mechanical, aerodynamic, manufacturing, and environmental requirements of composite materials for UAV micro-turbojet inlet applications.

### 2.7. Validation and Sensitivity Analysis

The robustness of the AHP-TOPSIS framework is validated through sensitivity analysis, examining the influence of weight variations on the final ranking of alternatives [16,17], the sensitivity analysis involves:

Scenario 1—Equal Weights: All criteria assigned equal importance to assess baseline performance

Scenario 2—Technical Priority: Higher weights assigned to mechanical and thermal criteria

Scenario 3—Environmental Priority: Higher weights assigned to sustainability criteria

Scenario 4—Economic Priority: Higher weights assigned to cost-related criteria

The ranking stability across scenarios indicates the robustness of the selected optimal material. Materials that consistently appear in the top positions across all scenarios are identified as robust alternatives suitable for the demanding conditions of UAV micro turbojet engine inlet applications [16,17].

## 3. Results

The MCDM (Multi-Criteria Decision-Making) analysis evaluated 3240 composite materials for use in the inlet zone of a UAV micro-turbojet engine. These composites were fabricated by combining

24 fibers (16 Natural + 8 Synthetic);

15 Matrices: Thermosets, Thermoplastics, and Biopolymers;

Vf Levels: 30%, 35%, 40%, 45%, 50%, 55%, 60%, 65%, and 70%;

Total Combinations: 3240 combinations.

The AHP-derived criteria weights presented in Table 7 were obtained through systematic pairwise comparisons of the ten evaluation criteria using Saaty’s fundamental 1–9 scale [16].

AHP is one of the most widespread MCDM methods. The advantage of the method is the ability to decompose the process under study.

The pairwise comparison matrix was constructed based on the operational requirements of UAV micro-turbojet engine inlet applications, where thermal and mechanical performance criteria were prioritized over economic and environmental considerations due to the demanding high-temperature, high-velocity flow conditions characteristic of the inlet section.

The AHP-derived criteria weights and rankings presented in Table 7 were obtained through a systematic and rigorous pairwise comparison process following Saaty’s fundamental 1–9 scale, specifically tailored to the operational demands of UAV micro-turbojet engine inlet applications. The pairwise comparison matrix was constructed by evaluating each criterion against every other criterion based on the functional requirements of the inlet zone, where materials must withstand high-temperature gas flow, aerodynamic pressures, and environmental exposure while maintaining structural integrity. For instance, Thermal Stability received the highest weight of 0.235 (23.5%) because it was judged as very strongly to extremely more important than Impact Strength (scored 4), Carbon Footprint (scored 6), and Biodegradability (scored 7), reflecting that continuous high-temperature exposure poses a persistent degradation threat that fundamentally determines material viability, whereas intermittent foreign object damage events and end-of-life considerations are secondary operational concerns. Similarly, Thermal Conductivity and Tensile Strength were assigned equal weights of 0.163 (16.3%) each, as both criteria are critical for inlet performance—thermal conductivity directly governs heat flux reduction and thermal gradient management, while tensile strength ensures structural integrity under aerodynamic pressure loading and mechanical stresses. The geometric mean method was subsequently employed to derive the normalized weight vector, where the geometric mean was computed for each criterion by multiplying the pairwise comparison values across each row and taking the nth root, followed by normalization to sum to unity. The resulting weights demonstrate a clear prioritization of technical performance criteria, with Thermal Stability (23.5%), Thermal Conductivity (16.3%), and Tensile Strength (16.3%) collectively accounting for 56.1% of the total weight, while Young’s Modulus, Flexural Strength, and Moisture Resistance each received 0.096 (9.6%), reflecting their important but secondary roles in ensuring dimensional stability, bending resistance, and protection against hygroscopic degradation in humid atmospheric conditions. Impact Strength received a moderate weight of 0.060 (6.0%), acknowledging its relevance for foreign object damage resistance while recognizing that FOD events are probabilistic and intermittent rather than continuous operational threats. In contrast, Material Cost (0.039, 3.9%), Carbon Footprint (0.029, 2.9%), and Biodegradability (0.022, 2.2%) received the lowest weights, confirming that economic viability and environmental sustainability are deprioritized relative to the demanding thermal and mechanical performance requirements of UAV inlet applications—a decision grounded in the engineering reality that material failure due to thermal degradation or structural compromise would result in catastrophic engine failure, mission loss, and potentially airframe destruction, far outweighing any cost savings or environmental benefits. The consistency ratio (CR) was calculated as 0.009, well below the acceptable threshold of 0.10, confirming that the pairwise comparisons were consistent and the derived weights are reliable for subsequent Multi-Criteria Decision-Making (MCDM) analysis. Overall, the AHP successfully translated the operational priorities of UAV inlet design into a quantitative weighting scheme that appropriately emphasizes thermal stability, thermal conductivity, and mechanical strength while appropriately de-emphasizing economic and environmental factors, thereby establishing a defensible and technically sound foundation for evaluating and ranking the 3240 composite material combinations under consideration.

The weighting scheme presented in Table 8 was developed through a systematic, scenario-based analytical approach designed to evaluate the suitability of Natural Fiber-Reinforced Polymer Composites (NFRPC) versus Synthetic Fiber-Reinforced Polymer Composites (SFRPC) specifically for UAV micro-turbojet inlet applications.

The foundation of this weighting framework rests on the Analytical Hierarchy Process (AHP) criteria identified in Table 8:

**Scenario 1 (Equal Weights)**, each of the ten criteria was assigned a uniform weight of 0.100, totalling 1.00. This baseline configuration serves as a neutral reference point that assumes all evaluation dimensions hold identical importance, allowing for an unbiased comparative assessment of NFRPC and SFRPC without any preconceived prioritization.

**Scenario 2 (Technical Priority)** reflects the operational reality that UAV inlets operate under extreme conditions where mechanical integrity and thermal management are paramount.

In this configuration, the highest weights were allocated to Thermal Stability (0.150) and Moisture Resistance (0.150), recognizing that inlet components must withstand elevated temperatures while maintaining dimensional stability in humid atmospheric conditions. Thermal Conductivity, Young’s Modulus, and Tensile Strength each received 0.130, emphasizing the critical importance of heat dissipation, structural stiffness under aerodynamic pressure, and load-bearing capacity for ensuring inlet geometry preservation and preventing catastrophic failure. Impact Strength (0.110) and Flexural Strength (0.090) received moderate weights, acknowledging their relevance for foreign object damage (FOD) resistance and bending loads while being slightly less critical than thermal and moisture-related properties.

Environmental and Economic criteria were deliberately suppressed, with Carbon Footprint (0.050) and Material Cost (0.050) receiving minimal weights, while Biodegradability was virtually disregarded at 0.010, reflecting the industry’s primary focus on performance and reliability over sustainability for high-performance military or commercial UAV applications.

**Scenario 3 (Environmental Priority)** Here, Carbon Footprint and Biodegradability receive the highest equal weights of 0.225 each, acknowledging that the aviation sector is increasingly scrutinized for its environmental impact and that UAV manufacturers may face future legislation requiring eco-friendly materials.

The Technical criteria are systematically downgraded, with Thermal Conductivity, Young’s Modulus, Tensile Strength, and Thermal Stability all receiving only 0.050—the lowest weights in this scenario, because environmentally focused designers may accept reduced performance in exchange for sustainability benefits, particularly for short-life or disposable UAV platforms. Impact Strength, Moisture Resistance, and Flexural Strength receive moderate weights of 0.075, while Material Cost at 0.075. This scenario effectively models the decision-making framework of organizations prioritizing operating under strict carbon accounting requirements.

**Scenario 4 (Economic Priority)** Material Cost receives the dominant weight of 0.225, reflecting the significant price differential between expensive carbon fiber and relatively inexpensive natural fibers. Interestingly, Thermal Conductivity, Young’s Modulus, Tensile Strength, and Thermal Stability are all assigned moderate weights of 0.075—reduced from their technical-priority levels but not entirely disregarded, because economic optimization cannot completely sacrifice performance without risking mission failure and associated financial losses. Impact Strength, Flexural Strength, and Moisture Resistance receive 0.100 each, balancing durability requirements with cost considerations, while Carbon Footprint (0.100) and Biodegradability (0.075) are moderately weighted to acknowledge that sustainability increasingly affects market access, brand value, and long-term regulatory compliance costs.

### 3.1. Top Performing Fibers from TOPSIS Rankings

The fiber ranking derived from the TOPSIS method is presented in Table 9. The fiber performance analysis reveals a stark contrast between synthetic and natural fibers, driven by the AHP criteria weights that heavily favor technical performance. This pronounced bias toward mechanical and thermal properties fundamentally shapes the ranking outcomes.

Table 9 shows that Synthetic fibers dominate the rankings, with all eight synthetic fibers occupying the top eight positions and achieving mean ranks ranging from 166.5 to 1295.4. Carbon (PAN-HM) emerges as the optimal fiber with a mean rank of 166.5 and TOPSIS score of 0.568. Carbon (Pitch-HP) and Carbon (Pitch-GP) follow closely, confirming carbon fibers as the superior choice for UAV inlet applications. Glass fibers (C-Glass and E-Glass) and Aramid fibers occupy the middle tier with TOPSIS scores of 0.299–0.329, offering good mechanical properties but with higher density (2.5–2.6 g/cm^3^) limiting their performance. In stark contrast, natural fibers occupy the bottom seven positions with mean ranks exceeding 1771 and TOPSIS scores below 0.135, demonstrating that despite their environmental advantages, their low thermal stability, high moisture absorption (7–15%), and significantly lower mechanical properties. Flax emerges as the best-performing natural fiber (mean rank: 1771.1, TOPSIS: 0.135), benefiting from its relatively high modulus (52 GPa) and low density (1.05 g/cm^3^), followed by Ramie and Pineapple.

The performance gap between synthetic and natural fibers is substantial the best natural fiber (Flax) achieves a TOPSIS score 4.2 times lower than the best synthetic fiber (Carbon PAN-HM).

### 3.2. Top Synthetic and Natural Fiber Composites

The results presented in Table 10 and Table 11 were derived from a systematic Multi-Criteria Decision-Making (MCDM) analysis employing the integrated Analytic Hierarchy Process (AHP) and Technique for Order Preference by Similarity to Ideal Solution (TOPSIS) framework, conducted at the optimal fiber fraction of Vf = 50%. The TOPSIS procedure involved normalizing the performance values using vector normalization, applying the AHP-derived criteria weights to construct a weighted normalized decision matrix, identifying the Positive Ideal Solution (PIS) and Negative Ideal Solution (NIS) for each criterion, and subsequently calculating the relative closeness coefficient for each composite alternative. The final ranking of all composite materials was then established by sorting the TOPSIS scores in descending order, where higher closeness coefficients indicate superior overall performance relative to the ideal solution.

The results in Table 10 and Table 11 reveal that synthetic fiber composites overwhelmingly dominate the top rankings, occupying all positions from Rank 1 through Rank 110. Table 11 shows that Carbon (PAN-HM) emerges as the superior fiber, achieving the highest TOPSIS score of 0.889312 when combined with Epoxy matrix, followed closely by Carbon (PAN-HM) with PC (0.888921) and Vinylester (0.875938). This exceptional performance is attributed to the outstanding thermal stability, exceptional tensile strength and ultra-low moisture absorption of PAN-based high-modulus carbon fibers, which align perfectly with the AHP-weighted priorities emphasizing thermal and mechanical performance.

Carbon (PAN-HM) demonstrates remarkable versatility by achieving high scores across diverse matrix types, including thermosets (Epoxy, Vinylester), thermoplastics (PC, ABS, PS, PET, PBT, Nylon 6), and even biopolymers (PLA, PHB), with TOPSIS scores ranging from 0.816 to 0.889. This indicates that the fiber properties dominate the composite performance, while the matrix serves primarily to transfer load and provide environmental protection.

Carbon (Pitch-HP) occupies ranks 10–15 with scores ranging from 0.805 to 0.822, slightly lower than PAN-HM. The consistent performance of thermoset matrices, particularly Epoxy, confirms their superior fiber-matrix interfacial adhesion and thermal stability compared to thermoplastics and biopolymers.

Table 11 demonstrates that natural fiber composites are relegated to ranks 111, with the best-performing natural composite.

Flax reinforced with Epoxy achieving a TOPSIS score of only 0.268588, which is approximately 3.3 times lower than the top synthetic composite (0.889312). Flax emerges as the best natural fiber due to its favorable combination of high modulus (52 GPa), low density (1.05 g/cm^3^), and relatively low moisture content (7.0%) among other natural fibers candidates.

The ranking pattern for natural fibers mirrors that of synthetics, with Epoxy consistently providing the best performance, followed by PC, confirming that thermoset matrices are optimal regardless of fiber type. Pineapple and Ramie occupy the next positions with scores of 0.262 and 0.259, respectively, benefiting from their high tensile strength and modulus. Kenaf, Banana, Corn, Jute, and Oil Palm follow with scores between 0.242 and 0.253, exhibiting moderate mechanical properties.

**Figure 3:**
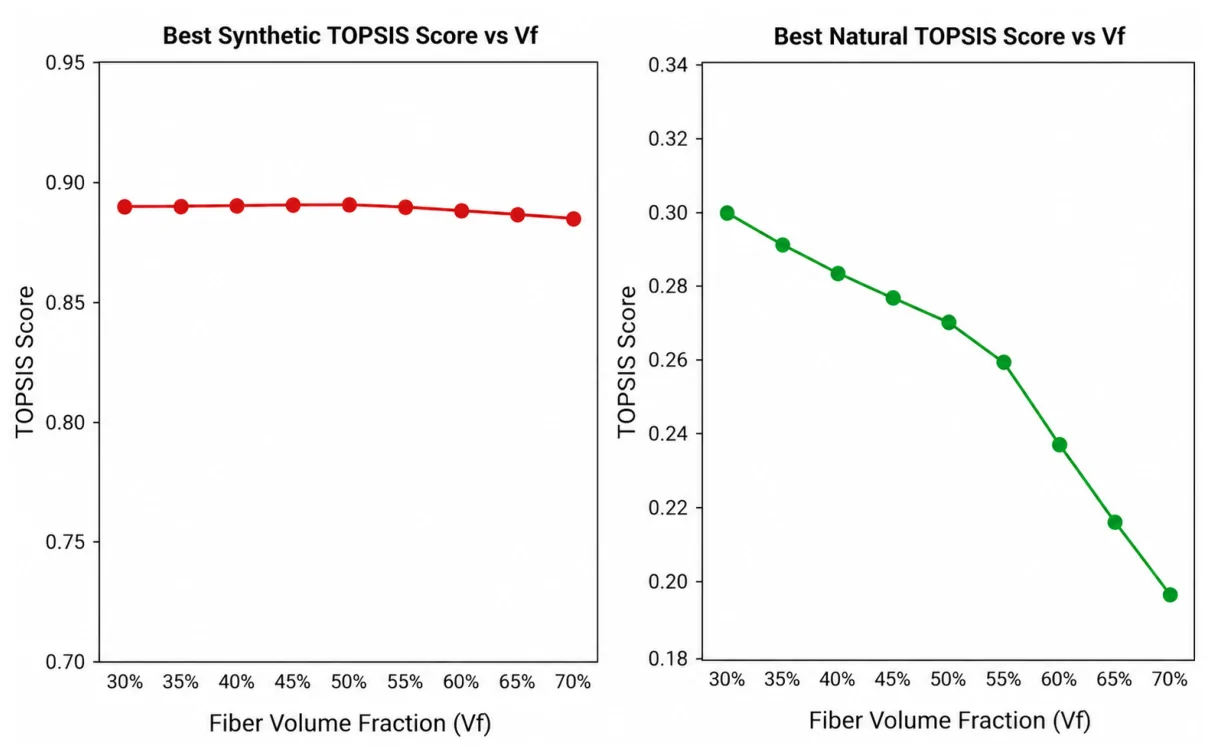
Effect of variation in Fiber Volume Fraction (Vf) on TOPSIS Scores for Optimal Synthetic and Natural Fiber Composites.

Figure 3 illustrating the effect of fiber volume fraction (Vf) on the TOPSIS scores for optimal synthetic and natural fiber composites was obtained through a systematic sensitivity analysis conducted as part of the integrated AHP-TOPSIS framework. The analysis evaluated 3240 composite material combinations across nine Vf levels ranging from 30% to 70% in 5% increments.

The results presented in Figure 3, shows the effect of Vf on composite performance.

The natural fiber composites exhibited a decreasing trend in TOPSIS scores as fiber volume fraction increased, with scores declining from approximately 0.30 at 30% Vf to approximately 0.20 at 70% Vf. This decline is attributed to the negative effects of increased moisture absorption and reduced thermal stability at higher Vf levels, which outweigh the mechanical property improvements gained from additional fiber content. In contrast, synthetic fiber composites maintained relatively stable TOPSIS scores across the entire Vf range (approximately 0.89 to 0.87), reflecting the superior thermal stability and moisture resistance of carbon fibers. The optimal Vf range for natural fiber composites is identified as 30–40%, where the best balance between mechanical performance and environmental resistance is achieved. For synthetic fiber composites, Vf = 50–55% provides the optimal balance between mechanical performance, manufacturability, and cost-effectiveness, consistent with aerospace industry practice.

### 3.3. Sensitivity Analysis Based on Scenarios

Based on the four scenarios defined in Table 8 and the optimal Vf = 50%, sensitivity analysis evaluates how the ranking of synthetic and natural fiber composites changes under different weighting priorities.

The selection of Vf = 50% as the representative fiber volume fraction for detailed material ranking was based on several considerations. First, Vf = 50% represents a practical fiber content for conventional composite manufacturing processes, including hand lay-up, vacuum bagging, and compression molding [35,36]. Second, at higher fiber volume fractions, achieving complete fiber wet-out and uniform matrix impregnation becomes more challenging, potentially increasing void content and reducing fiber–matrix interfacial quality [36,37]. Third, fiber volume fractions in the range of approximately 50% are commonly adopted in aerospace composite structures because they provide a balance between mechanical performance and manufacturing feasibility [38]. High fiber contents can increase manufacturing complexity and reduce impregnation quality [39]. Therefore, Vf = 50% was selected as the representative condition because it provides a practical balance between material performance, manufacturing feasibility, aerospace relevance, and consistency with the sensitivity-analysis results.

The results presented in Table 12 and Table 13 were obtained through a comprehensive scenario-based sensitivity analysis employing the integrated AHP-TOPSIS framework at the optimal fiber volume fraction of Vf = 50%. For each of the four weighting scenarios, the TOPSIS methodology was applied to evaluate all composite combinations (24 fibers × 15 matrices).

For each scenario, the decision matrix was normalized using vector normalization, the scenario-specific weights were applied to create a weighted normalized decision matrix, the Positive Ideal Solution (PIS) and Negative Ideal Solution (NIS) were identified, and the relative closeness coefficient was calculated for each alternative, enabling a comprehensive ranking of all composites under each weighting scenario. The top 10 Synthetic Fiber Composites on Table 12 and top 10 Natural Fiber Composites on Table 13.

The results presented in Table 12 and Table 13 demonstrate the robustness of the integrated AHP-TOPSIS framework under different weighting scenarios. Among the synthetic fiber composites, Carbon (PAN-HM) consistently maintained the highest ranking across all evaluation scenarios, indicating that its superior combination of mechanical strength, thermal stability, fatigue resistance, and low density makes it the most suitable reinforcement for UAV micro-turbojet engine inlet applications. This ranking stability confirms that the selection of Carbon (PAN-HM) is largely independent of decision-maker preferences and reflects its well-balanced engineering performance.

For the natural fiber composites, Flax consistently ranked as the highest-performing natural reinforcement in all scenarios. Although its overall performance remained below that of the leading synthetic fibers, its stable ranking demonstrates that flax offers the best balance of mechanical properties, lightweight characteristics, environmental performance, and processability among the natural fiber candidates evaluated. These findings identify flax as the most promising sustainable reinforcement for applications where environmental considerations are given greater importance.

## 4. Discussion

The results for all candidate fibers and matrices reveal the complete absence of any natural fiber composite in the top 110 rankings, confirming that natural fibers are unsuitable substitutes for synthetic fibers in high-performance UAV inlet applications that require thermal stability, structural integrity, and moisture resistance under high-velocity flow conditions. This performance disparity is fundamentally attributed to the inherent limitations of natural fibers, including their low thermal degradation threshold, high moisture absorption, and inferior mechanical properties compared to their synthetic counterparts. The AHP weighting scheme, which allocates most of the weight to technical criteria, inherently favors materials that excel in thermal and mechanical performance, further amplifying the advantage of synthetic fibers. However, the ranking methodology successfully identified the optimal natural fiber combinations for low-temperature, single-use, or environmentally prioritized UAV applications where sustainability considerations may partially outweigh technical performance requirements, with Flax + PLA emerging as the best natural composite under environmental weighting with a TOPSIS score of 0.5114.

### 4.1. Synthetic Fiber Composites Performance

The results for synthetic composites reveal several important observations regarding the ranking stability of synthetic fiber composites. Remarkably, Carbon (PAN-HM) remains the optimal synthetic fiber across all four scenarios, achieving the highest TOPSIS scores regardless of the weighting priorities, which confirms the exceptional and well-rounded performance of PAN-based high-modulus carbon fibers for UAV inlet applications. This consistency underscores the superior balance of properties offered by carbon fibers, including exceptional thermal stability (300 °C), ultra-high modulus (379.9 GPa), excellent tensile strength (2758 MPa), and negligible moisture absorption (0.1%). However, the optimal matrix selection demonstrates significant sensitivity to the weighting scenario. Under Scenario 1 (Equal), biopolymer matrices (PLA, PHB, PBS, PCL) dominate the top positions due to their balanced performance across all criteria, with Carbon (PAN-HM) + PLA achieving a score of 0.7981. In Scenario 2 (Technical), thermoset and thermoplastic matrices (Epoxy, PC, Vinylester, ABS, PS) dominate the top 10 positions, with Carbon (PAN-HM) + Epoxy achieving the highest overall score of 0.8718, reflecting the superior mechanical properties, thermal stability (Tg = 150 °C), and excellent fiber-matrix interfacial adhesion of epoxy matrices. In Scenario 3 (Environmental), biopolymer matrices overwhelmingly dominate all top 10 positions, with Carbon (PAN-HM) + PCL achieving the highest score of 0.6371, reflecting the exceptional biodegradability (9.8) and low carbon footprint (1.0) of PCL. Interestingly, Aramid I appears in the top 10 under the environmental scenario, demonstrating that even synthetic fibers with moderate environmental credentials can compete when sustainability is prioritized. In Scenario 4 (Economic), biopolymer matrices again dominate due to their lower material costs, with Carbon (PAN-HM) + PLA achieving a score of 0.7964. The performance gap between the best and worst synthetic composites across scenarios is substantial, ranging from 0.6371 (Environmental) to 0.8718 (Technical), confirming that weighting priorities significantly influence absolute scores, although the optimal fiber remains unchanged. This stability demonstrates that the AHP-TOPSIS framework is robust and reliable for material selection, with Carbon (PAN-HM) representing the undisputed optimal synthetic fiber for high-performance UAV inlet applications regardless of the specific design priorities.

### 4.2. Natural Fiber Composites Performance

For natural composites, the results reveal that Flax consistently emerges as the optimal natural fiber across all four scenarios, confirming its superior performance among natural fibers for UAV inlet applications. This consistency is attributed to Flax’s favorable combination of high modulus (52 GPa), low density (1.05 g/cm^3^), relatively high tensile strength (844 MPa), and moderate moisture content (7.0%) compared to other natural fibers. However, the optimal matrix selection for natural fibers demonstrates the same scenario-dependent behavior observed for synthetic fibers. Under Scenario 1 (Equal), biopolymer matrices (PLA, PCL, PHB, PBS) dominate all top 10 positions, with Flax + PLA achieving the highest score of 0.2937, reflecting the balanced performance of biopolymers across all criteria. In Scenario 2 (Technical), the ranking shifts dramatically, with thermoset and thermoplastic matrices (Epoxy, PC, Vinylester, ABS, PS, PET) occupying 9 of the top 10 positions, and Flax + Epoxy achieving the highest score of 0.2490, reflecting the superior mechanical properties and thermal stability of epoxy matrices. This represents a significant departure from the equal scenario, where biopolymers dominate. In Scenario 3 (Environmental), biopolymer matrices overwhelmingly dominate with all top 10 positions, and Flax + PLA achieving an impressive score of 0.5114, the highest natural fiber score across all scenarios, reflecting the exceptional biodegradability (9.5) and low carbon footprint (0.8) of flax combined with PLA’s environmental credentials. In Scenario 4 (Economic), biopolymer matrices again dominate with 9 of the top 10 positions, reflecting the lower material costs of biopolymers compared to thermosets and thermoplastics. The performance gap between the best and worst natural composites across scenarios is also substantial, ranging from 0.2490 (Technical) to 0.5114 (Environmental), confirming that weighting priorities significantly influence absolute scores. This indicates that while the optimal fiber remains stable, the optimal matrix is highly sensitive to the weighting scenario, with biopolymer matrices excelling under environmental and economic priorities, while thermosets dominate under technical priorities.

### 4.3. Performance Gap Analysis

The overall natural fiber scores (0.249–0.511) remain substantially lower than synthetic fiber scores (0.637–0.872), highlighting the fundamental performance limitations of natural fibers for demanding UAV inlet applications. This performance gap is primarily driven by the significantly higher AHP weights assigned to technical criteria, which favor synthetic fibers in domains where natural fibers are inherently deficient: thermal stability, mechanical strength, and moisture resistance. However, the environmental scenario narrows the gap significantly, with Flax + PLA achieving a score of 0.5114 compared to Carbon (PAN-HM) + PCL at 0.6371, representing a performance gap of only 24.6%. This suggests that for low-temperature, environmentally prioritized UAV applications where thermal stability and mechanical performance requirements are relaxed, natural fiber composites—particularly Flax reinforced with biopolymer matrices—could represent a viable and sustainable alternative to synthetic composites. The significant reduction in performance gap under environmental weighting (from 250.1% in Technical to 24.6% in Environmental) underscores the importance of aligning weighting priorities with application requirements. For single-use or disposable UAV platforms where operational lifespan is limited and environmental impact is a primary concern, natural fiber composites offer a compelling value proposition, combining acceptable performance with superior sustainability credentials. Conversely, for high-performance military or commercial UAV applications demanding reliability, durability, and performance under extreme conditions, synthetic fiber composites remain the only viable choice.

The sensitivity analysis confirms that the reinforcement ranking is robust, with Carbon (PAN-HM) consistently emerging as the optimal synthetic fiber and flax as the optimal natural fiber across all weighting scenarios. However, the preferred matrix material and absolute TOPSIS scores are sensitive to the selected priorities. Thermoset matrices such as epoxy dominate under technical weighting, while biopolymer matrices such as PLA, PCL, and PHB perform better under environmental and economic scenarios. Therefore, the robustness claim applies primarily to the fiber-level ranking, whereas the complete fiber–matrix composite ranking is more dependent on the selected scenario.

### 4.4. Comparison with Previous Studies

The application of MCDM methods to the selection and evaluation of fiber-reinforced composites for high-performance aerospace applications has been reported in several studies [40,41]. For example, Dewangan [40] applied an integrated AHP-TOPSIS approach to optimize the mechanical performance of jute–glass–carbon fiber-reinforced hybrid polymer composites. The study evaluated six stacking configurations under tensile and flexural loading and identified the carbon-rich cross-ply laminate as the best-performing configuration. This finding is consistent with the present study, in which carbon fiber-based composites achieved the highest rankings among the investigated synthetic fiber systems.

Similarly, Maity et al. [41] applied an MCDM approach to select a suitable material for the aerodynamic wing structure of a UAV. The study compared carbon fiber-reinforced polymer, Kevlar, balsa wood, polystyrene, and aluminum alloy and demonstrated the applicability of MCDM techniques for identifying materials that satisfy competing structural and sustainability requirements in UAV applications. The results support the use of a systematic multi-criteria framework for aerospace material selection.

The identification of flax as the highest-ranked natural fiber in the present study is also consistent with previous research on sustainable composites for UAV applications. Flax-based composites have demonstrated a favorable combination of specific mechanical properties and structural performance, supporting their potential for lightweight aerospace structures [37]. Furthermore, Gopinath Vinayagam et al. [2] investigated natural fiber composites for UAV microjet engine nozzles and demonstrated the potential of materials such as jute and hemp for applications involving relatively lower thermal demands. These findings are consistent with the present results, which indicate that natural fiber composites can provide viable alternatives under appropriate operating conditions but face limitations when high thermal and mechanical performance is required.

The overall ranking obtained in the present study, particularly the superior performance of carbon-fiber-based composites compared with natural-fiber systems, is also consistent with previous reviews of carbon composites for aerospace applications [42,43]. These studies highlight the high specific strength, stiffness, thermal resistance, and structural performance of carbon-based materials, which make them suitable for demanding aerospace applications.

The comparison with previous studies therefore shows good agreement between the proposed AHP-TOPSIS framework and established findings in aerospace composite-material selection. This agreement provides additional support for the reliability of the proposed methodology while also demonstrating its ability to incorporate thermal, mechanical, environmental, and economic criteria into a unified material-selection framework. The revised manuscript now includes this comparison to strengthen the validation and discussion of the obtained rankings.

### 4.5. Sustainability Implications of Material Selection

The study provides quantitative evidence of the sustainability trade-offs associated with composite material selection. The results show that the performance gap between synthetic and natural fiber composites decreases from 250.1% under the Technical weighting scenario to only 24.6% under the Environmental weighting scenario. This quantification enables designers to assess material options according to the specific sustainability priorities of each application.

Furthermore, the identification of flax-reinforced PLA and PCL composites as the best-performing natural fiber systems provides a potential pathway for reducing the environmental impact of UAV inlet materials. These biodegradable composite systems could offer an alternative to conventional synthetic composites where the required performance can be achieved with an acceptable reduction in mechanical or thermal performance.

The proposed framework also improves transparency in sustainability-oriented material selection by explicitly reporting the criteria, weighting factors, performance scores, and ranking procedure. This allows designers and other stakeholders to understand and justify material-selection decisions based on clearly defined priorities.

In addition, the identification of biodegradable fiber–matrix combinations supports circular-economy strategies by providing potential end-of-life options, such as industrial composting and anaerobic digestion, that are generally not available for conventional synthetic composite systems.

### 4.6. Limitations of the Study

Although this study provides a systematic framework for selecting composite materials for UAV inlet applications, several limitations should be recognized. The rule of mixtures used for estimating composite properties assumes ideal fiber–matrix bonding, uniform fiber distribution, and perfect fiber alignment. These assumptions may lead to an overestimation of the actual properties, particularly for natural fiber composites, where interfacial adhesion can be affected by fiber surface treatment, moisture content, fiber quality, and processing conditions.

The selected criteria cover the main thermal, mechanical, environmental, and economic properties considered in the present material-selection framework. However, several factors relevant to aerospace applications were not included, such as fatigue performance under cyclic loading, creep resistance at elevated temperatures, long-term environmental durability, ultraviolet resistance, fire and flame resistance, vibration damping, repairability, and joining and assembly requirements. The exclusion of these criteria was mainly due to the limited availability of consistent material-property data across all 3240 composite configurations.

In addition, the actual UAV inlet is exposed to combined service conditions, including cyclic thermal loading, aerodynamic pressure fluctuations, vibration, moisture, and other environmental effects. These conditions can interact and cause progressive degradation through mechanisms such as thermal aging, fatigue damage, moisture absorption, matrix degradation, and ultraviolet exposure. The present MCDM framework evaluates the available material properties independently and therefore does not fully capture these coupled degradation mechanisms.

The engine operating conditions used in this study were adopted from the experimental investigation reported by Balli [3]. The results should be interpreted within the operating range and conditions reported in that work. Further experimental investigations using different micro-turbojet engines and operating conditions would strengthen the generalizability of the present findings.

Consequently, the optimal candidates identified through the AHP-based MCDM analysis should be considered as promising materials rather than experimentally validated solutions for direct UAV inlet application. Further investigation is required through experimental characterization and numerical analysis under representative operating conditions. Future work should include thermal cycling, fatigue, creep, moisture and UV exposure, vibration, impact, and combined thermo-mechanical loading tests. Such validation will provide a more complete assessment of long-term durability and establish the suitability of the selected composite materials for practical UAV micro-turbojet inlet applications.

### 4.7. Future Research Directions

Future research should address the identified limitations by incorporating experimental validation of composite properties through comprehensive mechanical, thermal, and environmental testing of the optimal material combinations identified in this study. This should include tensile, flexural, and impact testing at both ambient and elevated temperatures, thermal cycling tests to evaluate dimensional stability and property retention, moisture absorption and desorption studies under representative environmental conditions, and accelerated aging tests to assess long-term durability and degradation mechanisms.

Additionally, comprehensive lifecycle assessment and lifecycle cost analysis should be conducted to quantify the full environmental and economic impacts of the candidate composites, considering raw material extraction, processing, manufacturing, operation, maintenance, and end-of-life disposal or recycling scenarios.

Experimental validation of selected materials under representative inlet operating conditions—including thermal cycling, moisture exposure, aerodynamic loading, and foreign object impact—is essential to confirm the predicted performance and identify any unforeseen failure mechanisms.

The methodology developed in this study provides a foundation for future investigations into sustainable composite materials for aerospace applications, supporting the transition toward more environmentally responsible UAV design while maintaining the high-performance standards required for safe and reliable operation.

## 5. Conclusions

This study successfully developed and applied an integrated AHP-TOPSIS framework for selecting optimal natural and synthetic fiber-reinforced polymer composites for UAV micro-turbojet engine inlet applications. The analysis evaluated 3240 composite combinations across ten harmonized criteria spanning technical, environmental, and economic dimensions, with AHP-derived normalized weights (CR = 0.009) confirming reliable pairwise comparisons. The TOPSIS analysis at the representative fiber volume fraction of Vf = 50% revealed that Carbon (PAN-HM) reinforced with Epoxy matrix is the unequivocally optimal composite, achieving the highest TOPSIS score of 0.8893.

In contrast, Flax + Epoxy emerged as the best natural composite with a score of only 0.2686, demonstrating a performance gap of approximately 231% in favor of synthetic fibers. Comprehensive sensitivity analysis across four weighting scenarios (Equal, Technical, Environmental, Economic) confirmed that the reinforcement ranking is highly robust, with Carbon (PAN-HM) remaining the optimal synthetic fiber and Flax the optimal natural fiber across all scenarios. However, the preferred matrix material and absolute TOPSIS scores exhibit sensitivity to the selected priorities: thermoset matrices (Epoxy) dominate under technical weighting, while biopolymer matrices (PLA, PCL, PHB) perform best under environmental and economic scenarios. The performance gap between synthetic and natural fibers varied considerably, ranging from 24.6% under Environmental weighting to 250.1% under Technical weighting, confirming that while environmental prioritization narrows the gap, it does not eliminate the fundamental performance limitations of natural fibers for demanding UAV inlet applications.

The Vf sensitivity analysis across 30% to 70% revealed that synthetic fiber composites maintain relatively stable performance across the entire Vf range (~0.89 to 0.87), while natural fiber composites exhibit a decreasing trend in TOPSIS scores with increasing Vf due to moisture absorption and thermal degradation effects. The optimal Vf range is identified as 30–40% for natural fiber composites and 50–55% for synthetic fiber composites, providing the best balance between mechanical performance, thermal stability, manufacturability, and cost-effectiveness.

Based on the comprehensive findings, the following recommendations are made for UAV inlet composite material selection:

For high-performance UAV applications requiring thermal stability and structural integrity, Carbon (PAN-HM) reinforced with Epoxy at Vf = 50–55% is strongly recommended as the optimal material combination.

For cost-sensitive applications, Carbon (Pitch-GP) with Epoxy or Vinylester offers a more economical alternative while maintaining acceptable performance.

For environmentally prioritized UAV applications, Flax reinforced with PLA or PCL biopolymer matrices at Vf = 30–40% represents the best natural fiber alternative.

For military, commercial, and high-reliability UAV platforms, synthetic fiber composites remain the only viable choice due to their superior long-term durability and thermal stability.

The optimal Vf should be selected based on manufacturing constraints and specific performance requirements, with Vf = 50–55% for synthetic composites and Vf = 30–40% for natural composites providing the best overall balance.

This study confirms that while natural fiber composites offer significant environmental advantages, they are currently unsuitable for high-performance UAV inlet applications that require thermal stability, structural integrity, and moisture resistance under high-velocity flow conditions. However, their potential for environmentally prioritized applications remains promising. The integrated AHP-TOPSIS framework provides a robust, transparent, and defensible methodology for composite material selection, enabling informed decision-making that balances technical performance, environmental sustainability, and economic viability.

Future research should include experimental validation of the identified optimal composites under representative inlet operating conditions, comprehensive lifecycle assessment and lifecycle cost analysis, investigation of hybrid natural-synthetic fiber composites, development of advanced fiber surface treatments, and exploration of additive manufacturing techniques for composite inlet fabrication, ultimately supporting the transition toward more environmentally responsible UAV design while maintaining the high-performance standards required for safe and reliable operation.

## Figures and Tables

**Table 1:** Basic measured data of MTJE at different operation modes [3].

Operation Mode	RPM	Air Flow Rate ṁ_1_ (kg/s)	Fuel Flow Rate ṁF (kg/s)	Exhaust Gas Rate ṁg (kg/s)	Thrust ET (N)	Exhaust Velocity V (m/s)
Mode-1	60,000	0.1324	0.00225	0.1347	45.04	334.5
Mode-2	80,000	0.1754	0.00273	0.1781	74.99	421.0
Mode-3	100,000	0.2075	0.00325	0.2108	100.00	474.5
Mode-4	120,000	0.2412	0.00388	0.2451	124.99	510.0

**Table 2:** Inlet conditions and performance parameters at Inlet Station [3].

Parameter	Symbol	Mode-1	Mode-2	Mode-3	Mode-4	Unit
Inlet Temperature	T_1_	288.15	288.15	288.15	288.15	K
Inlet Pressure	P_1_	101.325	101.325	101.325	101.325	kPa
Air Mass Flow Rate	ṁ_1_	0.1324	0.1754	0.2075	0.2412	kg/s
Specific Heat Capacity (Air)	cP,air	1.005	1.005	1.005	1.005	kJ/kg·K
Specific Gas Constant (Air)	R_air	0.287	0.287	0.287	0.287	kJ/kg·K
Reference Temperature	T_0_	288.15	288.15	288.15	288.15	K
Reference Pressure	P_0_	101.325	101.325	101.325	101.325	kPa

**Table 3:** Properties of Polymers Candidates.

Polymer Material	Elongation at Break (%)	Modulus of Elasticity (GPa)	Tensile Strength (MPa)	Density (g/cm^3^)	Tg (°C)	References
Non-biodegradable polymers						
Polycarbonate (PC)	70–150	2.0–2.44	60–72.4	1.14–1.21	149	[16,22]
Vinylester resin	2	2.0–4.5	40–90	1.20–1.50	50–150	[16,23]
Polystyrene (PS)	1–3.6	1.2–2.6	35.9–56.6	1.04–1.06	73.9–110	[16,24]
Epoxy	1–6	3.0–6.0	35–100	1.10–1.40	50–250	[11,16,25]
Polybutylene terephthalate (PBT)	250	1.93–3.0	50–60	1.30–1.38	40–60	[16,22]
Polyethylene terephthalate (PET)	30–300	2.76–4.14	48.3–72.4	1.29–1.40	70	[16,22]
Nylon 6	20–150	2.9	43–79	1.12–1.14	47	[16,26]
High-density polyethylene (HDPE)	2.0–130	0.4–1.5	14.5–38	0.94–0.96	−100	[16,22]
Low-density polyethylene (LDPE)	90–800	0.055–0.38	40–78	0.910–0.925	−100	[16,22]
Acrylonitrile butadiene styrene (ABS)	1.5–100	1.1–2.9	27.6–55.2	1.00–1.20	100	[16,22]
Polypropylene (PP)	15–700	0.95–1.77	26–41.4	0.899–0.920	−25	[16,22]
Biodegradable polymers						
PHB (Polyhydroxybutyrate)	3–6	1.7–3.5	40	1.25	5	[27]
PLA (Polylactic acid)	7–9	1.2–2.7	28–50	1.25	60–65	[27]
PCL (Polycaprolactone)	900	0.3	41	1.145	−60	[27]
PBS (Polybutylene succinate)	700	0.3–0.7	57	1.26	−32	[27,28]

**Table 4:** Properties of natural and synthetic fibers Candidates.

Fiber	Elongation at Break (%)	Young’s Modulus (GPa)	Tensile Strength (MPa)	Density (g/cm^3^)	Moisture Content (%)	References
Plant Natural Fibers						
Rice	2.2	0.3–2.6	19–135	1.4	7.9	[22]
Corn	3–4.7	10.1–16.3	355–580	1.2–1.4	8.5	[22]
Cotton	3–10	5.5–12.6	45.5–1000	1.5–1.6	9.8	[12,22]
Ramie	1.2–8	24.5–128	348–938	1.45–1.5	9	[22]
Kenaf	1.6–6.9	2.86–60	215.4–1191	0.6–1.5	9.2	[12,22]
Bamboo	1.5–11	11–17	140–230	0.6–11	8.9	[22]
Oil palm	8–25	1–9	92–1200	0.7–1.55	11	[22]
Flax	1.2–10	24–80	88–1600	0.6–1.5	7	[22]
Abaca	3–10	3–12	220–980	1.5	15	[22]
Banana	3–53	12–33.8	350–980	1.35	12.1	[22]
Jute	1.16–8	10–55	385–850	1.3–1.5	12	[22]
Pineapple	1–14.5	60–82	170–1672	0.8–1.6	13	[22]
Sisal	2–25	9–38	80–840	1.3–1.5	11	[22]
Coir	14.21–59.9	1.27–6	106–593	1.1–1.6	10	[22]
Coconut	10–23	21.1	150	0.43	8.2	[22]
Sugarcane bagasse	1.1	17–27.1	20–290	1.2–1.5	8.8	[22]
Synthetic Fibers						
Acrylic	7.5–50	13.79–19.31	269–1000	1.16–1.18	-	[29]
Aramid I	4.4	62.06	2930	1.44	-	[29]
Aramid II	2.5	117.2	2344	1.44	-	
Carbon (PAN-HM)	0.5–0.7	379.9	2482–3034	1.6–1.7	-	[29,30]
Carbon (Pitch-GP)	2–2.4	27.58–34.48	483–793	1.6–1.7	-	[29]
Carbon (Pitch-HP)	0.5–1.1	151.7–482.7	1517–3103	1.8–2.15	-	[29]
C-Glass	4.8	68.9	3310	2.52	-	[27]
E-Glass	1.8–4.8	70–76	2000–3500	2.5–2.59	-	[27]

**Table 5:** The dimensions and the criteria of the study.

Dimension	Criteria	Type
Environmental	CO_2_ footprint, waste diversion, toxicity, biodegradability	Benefit
Economic	Material cost, processing cost, lifecycle cost	Cost
Technical	Tensile strength, flexural strength, impact strength, hardness, thermal conductivity, thermal stability	Benefit

**Table 6:** AHP-derived criteria weights for UAV inlet material selection.

Dimension	Criterion	Justification
Technical	Thermal Conductivity	Primary driver for heat flux reduction
Technical	Young’s Modulus	Determines stiffness and dimensional stability under aerodynamic pressure
Technical	Tensile Strength	Critical for structural integrity under loading
Technical	Thermal Stability	Essential for high-temperature operation
Technical	Impact Strength	Resistance to foreign object damage
Environmental	Carbon Footprint	Sustainability and emissions reduction
Economic	Material Cost	Economic viability and lifecycle cost
Technical	Flexural Strength	Bending resistance under operational loads
Technical	Moisture resistance	Prevents hygroscopic degradation, delamination, and property loss in humid/rainy operating environments
Environmental	Biodegradability	End-of-life environmental impact

**Table 7:** AHP-Derived Criteria Weights and Rankings.

Criterion	AHP Weight	Normalized Weight (%)	Interpretation
Thermal Stability	0.235	23.5%	Most critical criterion; inlet material must withstand continuous high-temperature gas flow without degradation.
Thermal Conductivity	0.163	16.3%	Second most important; high thermal conductivity reduces thermal gradients and heat accumulation.
Tensile Strength	0.163	16.3%	Equally critical as thermal conductivity; ensures structural integrity under pressure and aerodynamic loading.
Young’s Modulus	0.096	9.6%	Important for dimensional stability; prevents inlet geometry deformation under ram air pressure.
Flexural Strength	0.096	9.6%	Equivalent to Young’s Modulus; resists bending loads from aerodynamic forces.
Moisture Resistance	0.096	9.6%	Essential for preventing hygroscopic degradation in humid atmospheric conditions.
Impact Strength	0.060	6.0%	Moderately important; provides resistance to FOD but less critical than thermal/structural properties.
Material Cost	0.039	3.9%	Low priority; economic considerations are secondary to performance and safety.
Carbon Footprint	0.029	2.9%	Very low priority; sustainability is deprioritized relative to technical requirements.
Biodegradability	0.022	2.2%	Lowest priority; end-of-life environmental impact is negligible compared to operational performance.
Total	1.000	100%	

**Table 8:** Assigned Criteria Weights for Each Scenario.

Criterion	Scenario 1 (Equal)	Scenario 2 (Technical)	Scenario 3 (Environmental)	Scenario 4 (Economic)
Thermal Conductivity	0.100	0.130	0.050	0.075
Young’s Modulus	0.100	0.130	0.050	0.075
Tensile Strength	0.100	0.130	0.050	0.075
Thermal Stability	0.100	0.150	0.050	0.075
Impact Strength	0.100	0.110	0.075	0.100
Carbon Footprint	0.100	0.050	0.225	0.100
Material Cost	0.100	0.050	0.075	0.225
Flexural Strength	0.100	0.090	0.075	0.100
Moisture Resistance	0.100	0.150	0.075	0.100
Biodegradability	0.100	0.010	0.225	0.075
Total	1.00	1.00	1.00	1.00

**Table 9:** Top 15 Performing Fibers from TOPSIS Rankings.

Rank	Fiber	Mean Rank	TOPSIS Score Mean
1	Carbon (PAN-HM)	166.489	0.568
2	Carbon (Pitch-HP)	203.867	0.538
3	Carbon (Pitch-GP)	415.415	0.392
4	C-Glass	563.481	0.329
5	Aramid I	615.081	0.313
6	E-Glass	662.407	0.299
7	Aramid II	693.237	0.290
8	Acrylic	1295.385	0.170
9	Flax	1771.126	0.135
10	Ramie	1932.370	0.125
11	Pineapple	1936.748	0.125
12	Kenaf	2008.674	0.120
13	Corn	2131.711	0.113
14	Banana	2186.052	0.109
15	Cotton	2187.341	0.110

**Table 10:** Top 15 Synthetic Fiber Composites at Vf = 50%.

Rank	Fiber	Matrix	Matrix Type	TOPSIS Score
1	Carbon (PAN-HM)	Epoxy	Thermoset	0.889312
2	Carbon (PAN-HM)	PC	Thermoplastic	0.888921
3	Carbon (PAN-HM)	Vinylester	Thermoset	0.875938
4	Carbon (PAN-HM)	ABS	Thermoplastic	0.872726
5	Carbon (PAN-HM)	PS	Thermoplastic	0.869567
6	Carbon (PAN-HM)	PET	Thermoplastic	0.859261
7	Carbon (PAN-HM)	PLA	Biopolymer	0.856473
8	Carbon (PAN-HM)	PBT	Thermoplastic	0.846149
9	Carbon (PAN-HM)	Nylon 6	Thermoplastic	0.844819
10	Carbon (Pitch-HP)	Epoxy	Thermoset	0.822372
11	Carbon (Pitch-HP)	PC	Thermoplastic	0.821096
12	Carbon (PAN-HM)	PHB	Biopolymer	0.816476
13	Carbon (Pitch-HP)	Vinylester	Thermoset	0.811341
14	Carbon (Pitch-HP)	ABS	Thermoplastic	0.807492
15	Carbon (Pitch-HP)	PS	Thermoplastic	0.805430

**Table 11:** Top 15 Natural Fiber Composites at Vf = 50%.

Rank	Fiber	Matrix	Matrix Type	TOPSIS Score
111	Flax	Epoxy	Thermoset	0.268588
112	Flax	PC	Thermoplastic	0.267023
114	Pineapple	Epoxy	Thermoset	0.261996
115	Pineapple	PC	Thermoplastic	0.260290
116	Ramie	Epoxy	Thermoset	0.259357
117	Ramie	PC	Thermoplastic	0.257626
119	Kenaf	Epoxy	Thermoset	0.253114
120	Kenaf	PC	Thermoplastic	0.251628
123	Banana	Epoxy	Thermoset	0.243610
124	Corn	Epoxy	Thermoset	0.243589
125	Jute	Epoxy	Thermoset	0.243174
126	Corn	PC	Thermoplastic	0.242210
127	Banana	PC	Thermoplastic	0.242134
128	Oil Palm	Epoxy	Thermoset	0.242077
129	Jute	PC	Thermoplastic	0.241651

**Table 12:** Top 10 Synthetic Fiber Composites by Scenario at Vf = 50%.

Rank	Scenario 1 (Equal)	Score	Scenario 2 (Technical)	Score	Scenario 3 (Environmental)	Score	Scenario 4 (Economic)	Score
1	Carbon (PAN-HM) + PLA	0.7981	Carbon (PAN-HM) + Epoxy	0.8718	Carbon (PAN-HM) + PCL	0.6371	Carbon (PAN-HM) + PLA	0.7964
2	Carbon (PAN-HM) + PHB	0.7880	Carbon (PAN-HM) + PC	0.8709	Carbon (PAN-HM) + PLA	0.6366	Carbon (PAN-HM) + PHB	0.7926
3	Carbon (PAN-HM) + PBS	0.7816	Carbon (PAN-HM) + Vinylester	0.8661	Carbon (PAN-HM) + PHB	0.6276	Carbon (PAN-HM) + PBS	0.7852
4	Carbon (PAN-HM) + PCL	0.7785	Carbon (PAN-HM) + ABS	0.8634	Carbon (PAN-HM) + PBS	0.6256	Carbon (PAN-HM) + PCL	0.7832
5	Carbon (Pitch-HP) + PLA	0.7475	Carbon (PAN-HM) + PS	0.8623	Carbon (Pitch-HP) + PCL	0.6244	Carbon (PAN-HM) + Epoxy	0.7584
6	Carbon (Pitch-HP) + PHB	0.7391	Carbon (PAN-HM) + PET	0.8587	Carbon (Pitch-HP) + PLA	0.6239	Carbon (PAN-HM) + Vinylester	0.7528
7	Carbon (PAN-HM) + Epoxy	0.7385	Carbon (PAN-HM) + PLA	0.8546	Carbon (Pitch-HP) + PHB	0.6148	Carbon (PAN-HM) + PC	0.7525
8	Carbon (PAN-HM) + PC	0.7376	Carbon (PAN-HM) + PBT	0.8528	Carbon (Pitch-HP) + PBS	0.6128	Carbon (Pitch-HP) + PLA	0.7521
9	Carbon (PAN-HM) + Vinylester	0.7357	Carbon (PAN-HM) + Nylon 6	0.8528	Aramid I + PLA	0.5254	Carbon (Pitch-HP) + PHB	0.7496
10	Carbon (Pitch-HP) + PBS	0.7332	Carbon (PAN-HM) + PHB	0.8369	Aramid I + PCL	0.5250	Carbon (PAN-HM) + Nylon 6	0.7483

**Table 13:** Top 10 Natural Fiber Composites by Scenario at Vf = 50%.

Rank	Scenario 1 (Equal)	Score	Scenario 2 (Technical)	Score	Scenario 3 (Environmental)	Score	Scenario 4 (Economic)	Score
1	Flax + PLA	0.2937	Flax + Epoxy	0.2490	Flax + PLA	0.5114	Flax + PLA	0.2773
2	Flax + PCL	0.2856	Flax + PC	0.2466	Flax + PCL	0.5108	Flax + PHB	0.2745
3	Flax + PHB	0.2856	Ramie + Epoxy	0.2345	Flax + PHB	0.5071	Flax + PCL	0.2727
4	Flax + PBS	0.2835	Ramie + PC	0.2316	Pineapple + PLA	0.5066	Flax + PBS	0.2703
5	Pineapple + PLA	0.2809	Flax + Vinylester	0.2314	Pineapple + PCL	0.5061	Flax + Epoxy	0.2589
6	Ramie + PLA	0.2790	Flax + ABS	0.2287	Flax + PBS	0.5047	Ramie + PLA	0.2583
7	Pineapple + PCL	0.2724	Flax + PS	0.2265	Sugarcane + PLA	0.5027	Ramie + PHB	0.2550
8	Pineapple + PHB	0.2722	Flax + PET	0.2217	Sugarcane + PCL	0.5024	Pineapple + PLA	0.2538
9	Kenaf + PLA	0.2716	Flax + PLA	0.2175	Pineapple + PHB	0.5021	Ramie + PCL	0.2531
10	Ramie + PHB	0.2703	Pineapple + Epoxy	0.2170	Banana + PLA	0.5020	Kenaf + PLA	0.2515

## Data Availability

The original contributions presented in this study are included in the article. Further inquiries can be directed at the corresponding author.

## Abbreviations

NFRPCs	Natural fiber-reinforced polymer composites
SFRPCs	Synthetic fiber-reinforced polymer composites
AHP	Analytic Hierarchy Process
TOPSIS	Technique for Order Preference by Similarity to Ideal Solution
MCDM	multi-criteria decision-making
MTJE	Micro-turbojet engine
